# Music Learning Self-Efficacy and Deliberate Music Practice: The Mediating Role of Achievement Goal Orientation and the Moderating Role of AI Literacy

**DOI:** 10.3390/bs16081456

**Published:** 2026-08-21

**Authors:** Shihan Wang, Ziqiao Wang, Baoqian Yang, Lifang Tang

**Affiliations:** 1 Faculty of Education, Northeast Normal University, 5268 Renmin Street, Changchun 130024, China; wangshihan@nenu.edu.cn (S.W.);; 2 School of Teacher Education, Yanbian University, 977 Gongyuan Road, Yanji 133002, China

**Keywords:** music learning self-efficacy, deliberate music practice, achievement goal orientation, AI literacy, moderated mediation

## Abstract

Deliberate practice plays a central role in high-quality music learning, yet the motivational and technology-related factors associated with sustained deliberate music practice remain insufficiently understood. Drawing on social cognitive theory, achievement goal theory, and the literature on artificial intelligence (AI) literacy, this study examined the relationships among music learning self-efficacy, achievement goal orientation, AI literacy, and deliberate music practice. A total of 458 music students from a teacher-training university in Liaoning Province, China, completed measures of the four constructs. Music learning self-efficacy was positively associated with deliberate music practice. Achievement goal orientation also showed a significant indirect association between music learning self-efficacy and deliberate music practice, such that students with higher self-efficacy reported stronger achievement goal orientations, which in turn were associated with greater engagement in deliberate music practice. AI literacy further moderated the association between achievement goal orientation and deliberate music practice, with this positive relationship being stronger among students reporting higher levels of AI literacy. These findings suggest that deliberate music practice is associated with both learners’ motivational beliefs and their self-reported AI literacy.

## 1. Introduction

Music learning depends heavily on sustained practice because learners must continually refine technical control, auditory judgment, musical understanding, and expressive performance (Jørgensen & Hallam, 2009). Yet practice time alone does not fully account for differences in musical development. Learners who spend similar amounts of time practising may achieve markedly different outcomes depending on how that practice is structured and regulated (Duke et al., 2009; Macnamara et al., 2014). Deliberate practice emphasizes purposeful, feedback-informed, and strategically adjusted activity rather than mere repetition (Ericsson et al., 1993; Ericsson, 2006). In music learning, such practice typically involves setting specific goals, identifying performance problems, selecting appropriate strategies, monitoring progress, and breaking difficult tasks into manageable components (Chaffin & Imreh, 2001; Passarotto et al., 2022).

Research on effective music practice has highlighted the importance of metacognitive monitoring, self-regulation, problem identification, strategic planning, and process evaluation (Hallam, 2001; McPherson & Renwick, 2001). Related studies have shown that skilled learners differ in how they organize, monitor, and adapt their practice activities over time (Miksza et al., 2012; Nielsen, 2001). This literature has substantially clarified the characteristics of effective practice, but it has offered less explanation of the motivational processes that make some learners more likely than others to engage in deliberate practice consistently (Miksza, 2011a). Competence beliefs and achievement goals are likely to be especially relevant because they shape how learners interpret difficulty, allocate effort, persist with challenging tasks, and regulate learning strategies (Bandura, 1986; Pintrich, 2000; Schunk & DiBenedetto, 2020). Examining these processes may therefore extend music practice research from descriptions of effective behavior toward an explanation of the psychological conditions associated with such behavior.

Music learning self-efficacy provides a useful starting point for understanding these differences. Self-efficacy refers to individuals’ beliefs about their capability to organize and execute the actions required to attain desired outcomes and has been consistently associated with effort, persistence, task choice, and strategic regulation (Bandura, 1977, 1997). In music education, self-efficacy has been examined primarily in relation to performance achievement and performance-related outcomes (McCormick & McPherson, 2003; McPherson & McCormick, 2006). Research on measurement has further emphasized that efficacy beliefs are most meaningful when assessed within specific domains of musical learning and performance (Ritchie & Williamon, 2011; Zelenak, 2015). Comparatively less attention has been directed toward the practice processes through which musical performance is developed. Because progress in music depends on repeated engagement with specific learning problems, self-efficacy may be particularly relevant to whether learners persist with demanding practice tasks and regulate them strategically (McPherson & Zimmerman, 2011).

The increasing use of artificial intelligence (AI) adds a further layer to this motivational process by changing the kinds of informational and instructional resources available during practice. AI-based tools can provide access to information, support musical analysis and creative work, and offer forms of personalized assistance in music learning (Holland, 2000; Mazlan et al., 2026). At the same time, their educational value is not automatic. Concerns have been raised about inappropriate use of feedback, overreliance on automated systems, and difficulties in integrating AI meaningfully into pedagogical practice (Merchán Sánchez-Jara et al., 2024). Similar opportunities and limitations have been identified in broader educational settings (Holmes et al., 2019; Luckin et al., 2016). These findings suggest that the usefulness of AI-supported learning may depend partly on whether learners can interpret, evaluate, and apply AI-generated information appropriately (Zawacki-Richter et al., 2019; Casal-Otero et al., 2023).

AI literacy captures this capacity by encompassing learners’ understanding of AI, their ability to use AI applications, their evaluation of AI-generated outputs, and their awareness of ethical issues surrounding AI use (Long & Magerko, 2020; Wang et al., 2023). Such competencies may be particularly relevant in music practice, where learners increasingly encounter AI-generated feedback, recommendations, and learning resources that require active judgment rather than passive acceptance (Ng et al., 2021). Existing research in music education has largely focused on the potential applications of AI and the pedagogical challenges associated with its use (Cheng, 2025; Merchán Sánchez-Jara et al., 2024). Much less is known about whether differences in learners’ AI literacy are associated with the way they engage in deliberate practice. It also remains unclear whether the relationship between motivational orientations and high-quality practice behaviors varies across levels of AI literacy (Mazlan et al., 2026).

Against this background, the present study examines a second-stage moderated mediation model linking music learning self-efficacy, achievement goal orientation, AI literacy, and deliberate music practice. Achievement goal orientation is considered a motivational mechanism through which competence beliefs may be associated with deliberate practice (Elliot & McGregor, 2001), whereas AI literacy is examined as a boundary condition that may alter the strength of the association between achievement goal orientation and deliberate practice. By integrating motivational beliefs with learners’ capacity to engage with AI-supported learning resources, the study seeks to clarify why some music learners are more likely to engage in deliberate practice and under what conditions this association may be stronger.

### 1.1. Music Learning Self-Efficacy and Deliberate Music Practice

Music learning self-efficacy refers to learners’ beliefs in their capability to successfully manage music learning tasks, acquire musical skills, and meet the demands of practice and performance (Ritchie & Williamon, 2011). Within social cognitive theory, self-efficacy represents a domain- and task-specific competence belief that shapes task choice, effort, persistence, and self-regulatory behavior (Bandura, 1997). Learners with stronger efficacy beliefs are generally more willing to undertake challenging tasks and sustain effort when difficulties arise (Pajares, 1996). These tendencies are particularly relevant to music learning, where effective practice requires ongoing problem solving, error correction, strategic decision making, and self-regulation (McPherson & Zimmerman, 2011).

Deliberate music practice involves purposeful goal setting, the use of feedback, identification of performance problems, and continuous strategic adjustment (Ericsson et al., 1993; Passarotto et al., 2022). Effective practice likewise depends on learners’ ability to diagnose errors, monitor progress, modify strategies, and move beyond mechanical repetition (Duke et al., 2009; Hallam, 2001). From a social cognitive perspective, learners who are more confident in their ability to overcome difficult passages or master complex tasks should be more likely to persist when problems emerge and to regulate their practice strategically (Schunk & DiBenedetto, 2020). In contrast, weaker efficacy beliefs may be associated with reduced persistence, avoidance of difficult material, and less systematic engagement with practice-related problems (Nielsen, 2001).

Previous research in music education has linked self-efficacy primarily to performance achievement and other performance-related outcomes (McCormick & McPherson, 2003; McPherson & McCormick, 2006). At the same time, research on musical self-efficacy has emphasized the importance of examining efficacy beliefs in relation to specific learning tasks and contexts (Ritchie & Williamon, 2011). Comparatively less attention has been given to the role of music learning self-efficacy in the quality of practice that precedes performance. This is an important distinction because musical development depends not only on eventual performance outcomes but also on how learners regulate, adapt, and sustain their practice over time (McPherson & Zimmerman, 2011).

Focusing on deliberate music practice therefore extends research on musical self-efficacy from performance outcomes to the learning processes through which those outcomes are developed. It also addresses the limited attention given to the motivational factors associated with learners’ engagement in high-quality practice behaviors (Miksza, 2011a). Consistent with social cognitive theory, stronger music learning self-efficacy is expected to be associated with greater engagement in deliberate, strategically regulated practice (Bandura, 1997; Passarotto et al., 2022). Accordingly, the following hypothesis is proposed:

**H1.**  *Music learning self-efficacy is positively associated with deliberate music practice.*

### 1.2. The Mediating Role of Achievement Goal Orientation

Although music learning self-efficacy reflects learners’ beliefs about their capability to manage music learning tasks, the association between such beliefs and deliberate practice may involve additional motivational processes (Bandura, 1986). Social cognitive theory suggests that efficacy beliefs influence learning behavior partly through their effects on goal setting, self-regulation, and behavioral choices (Bandura, 1997). Achievement goal orientation is therefore relevant because it concerns how learners define competence, interpret success and failure, and regulate their behavior in achievement settings (Ames, 1992; Schunk & DiBenedetto, 2020).

Achievement goal theory conceptualizes goal pursuit as multidimensional. Earlier work differentiated major motivational orientations toward competence and achievement (Dweck & Leggett, 1988; Elliot & Church, 1997), while the 2 × 2 framework distinguished mastery and performance goals along approach and avoidance dimensions (Elliot & McGregor, 2001). The Achievement Goal Questionnaire-Revised was subsequently developed to assess these distinct goal orientations (Elliot & Murayama, 2008), and later reviews have continued to emphasize the multidimensional nature of achievement goals across educational contexts (Hulleman et al., 2010; Scherrer et al., 2020). While these distinctions remain theoretically important, the present study focuses on learners’ broader achievement-related motivational orientation within the proposed mediation model rather than on contrasts among individual goal dimensions. The overall score is therefore used to represent the extent to which learners organize their music learning around achievement- and competence-related goals. The empirical suitability of this representation was additionally evaluated through confirmatory factor analyses reported in the measurement results.

Music learning self-efficacy may be associated with achievement goal orientation because beliefs about capability can shape the kinds of goals learners are willing to adopt and pursue (Bandura, 1997). Learners who feel capable of mastering difficult musical tasks may be more willing to approach technical challenges as attainable goals and to sustain their efforts toward improvement (Pajares, 1996). Consistent with this reasoning, self-efficacy has been associated with achievement goal orientations and adaptive patterns of goal pursuit in educational settings (Alhadabi & Karpinski, 2020). Goal orientation has also been identified as a motivational process linking efficacy beliefs with learning strategies and academic outcomes (Honicke et al., 2020; Zhong et al., 2023).

Achievement goal orientation may, in turn, be related to deliberate music practice because learners’ goals influence how they allocate effort, select strategies, and evaluate their progress (Pintrich, 2000). Learners with stronger achievement-related goal orientations may be more likely to remain engaged when difficulties arise and to modify their strategies in response to performance problems (Senko et al., 2011). These behaviors are closely aligned with the goal-directed and self-regulated nature of deliberate practice (Passarotto et al., 2022). Effective music practice likewise depends on identifying problems, monitoring progress, and making strategic adjustments over time (Hallam, 2001), while achievement goal motivation has been linked to practice behaviors among collegiate instrumentalists (Miksza, 2011b).

Taken together, these relationships support a potential indirect pathway in which music learning self-efficacy is associated with stronger achievement goal orientation, which is subsequently related to greater engagement in deliberate music practice. Such a pathway is theoretically plausible because deliberate practice requires learners to sustain goal-directed effort and continually adapt their behavior in response to feedback and performance demands (Ericsson et al., 1993). Examining achievement goal orientation as an intervening motivational process may therefore help connect research on self-efficacy and music performance outcomes (McPherson & McCormick, 2006) with research on the motivational foundations of high-quality practice behavior (Miksza, 2011a; Honicke et al., 2020).

Based on the above theory and research, the following hypothesis is proposed:

**H2.**  *Achievement goal orientation mediates the relationship between music learning self-efficacy and deliberate music practice.*

### 1.3. The Moderating Role of AI Literacy

Achievement goal orientation provides direction for learners’ effort and engagement, but the strength of its association with deliberate practice may vary across learning conditions (Pintrich, 2000). Deliberate music practice requires learners to identify problems, select appropriate strategies, interpret feedback, and continually adapt their practice activities (Ericsson et al., 1993). Because music practice involves complex demands related to technical control, auditory judgment, musical understanding, and expressive performance, the extent to which achievement goals are associated with deliberate practice may also depend on learners’ access to relevant learning resources and their capacity to use those resources effectively (Bandura, 1986; Jørgensen & Hallam, 2009).

AI has become an increasingly relevant learning resource in music education, offering support for information retrieval, musical analysis, creative activity, personalized learning, and feedback generation (Mazlan et al., 2026; Merchán Sánchez-Jara et al., 2024). The educational value of such resources, however, depends partly on how learners interpret and use them. Access to AI alone does not ensure effective learning support, particularly when learners differ in their ability to evaluate the accuracy, relevance, and limitations of AI-generated information (Zawacki-Richter et al., 2019). Previous research has also identified concerns related to inappropriate feedback use, limited understanding of AI systems, ethical issues, and difficulties in pedagogical integration (Chiu et al., 2023). These challenges make AI literacy particularly relevant to learning in AI-supported environments (Ng et al., 2021).

AI literacy refers to learners’ capacity to understand AI, use AI applications, critically evaluate AI-generated outputs, and engage with AI in an informed and ethically responsible manner (Wang et al., 2023). It also involves recognizing the capabilities and limitations of AI systems and judging whether their outputs are reliable and appropriate for a particular learning purpose (Long & Magerko, 2020). In music learning, stronger AI literacy may allow learners to engage more critically with AI-supported resources when analyzing musical structures, considering practice suggestions, or comparing possible learning strategies (Mazlan et al., 2026). It may also enable them to evaluate, adapt, or reject AI-generated feedback when necessary rather than accepting it uncritically (Ng et al., 2021).

From this perspective, AI literacy may serve as a boundary condition in the association between achievement goal orientation and deliberate music practice. Learners with stronger achievement goal orientations are generally more inclined to organize their learning around competence development and goal attainment (Elliot & McGregor, 2001). When AI literacy is higher, these learners may be better equipped to draw on AI-supported resources for planning practice, evaluating feedback, and refining learning strategies. Their achievement-related motivation may therefore be more strongly associated with deliberate practice under conditions of higher AI literacy (Wang et al., 2023; Chiu et al., 2023).

This proposition extends existing explanations of deliberate music practice, which have largely emphasized goal setting, problem identification, strategic adjustment, and self-monitoring (Duke et al., 2009; Passarotto et al., 2022). Technological competence has received comparatively less attention as a factor that may shape how motivational orientations are expressed in music practice behavior (Miksza, 2011a). Although recent research has begun to examine the applications and challenges of AI in music education, little is known about whether individual differences in AI literacy alter the association between achievement goals and deliberate practice (Cheng, 2025; Merchán Sánchez-Jara et al., 2024). AI literacy may therefore help explain when achievement goal orientation is more strongly associated with deliberate music practice in increasingly AI-supported learning environments (Ng et al., 2021).

Based on the above theory and research, the following hypothesis is proposed:

**H3.**  *AI literacy moderates the relationship between achievement goal orientation and deliberate music practice. Specifically, the positive relationship between achievement goal orientation and deliberate music practice is stronger when AI literacy is higher.*

**H4.**  *The indirect relationship between music learning self-efficacy and deliberate music practice through achievement goal orientation varies as a function of AI literacy. Specifically, this indirect relationship is stronger among learners with higher AI literacy.*

### 1.4. The Present Study

Taken together, previous research has established that high-quality music practice involves goal setting, problem identification, feedback use, and strategic adjustment (Ericsson et al., 1993; Passarotto et al., 2022), yet less is known about the psychological mechanisms associated with learners’ engagement in such practice (Miksza, 2011a). Research on musical self-efficacy has focused largely on performance and learning outcomes, with comparatively less attention paid to the practice process preceding performance (McPherson & McCormick, 2006; Ritchie & Williamon, 2011). Meanwhile, emerging research on AI in music education has examined the educational value and pedagogical challenges of AI tools but has rarely considered how learners’ own AI literacy may condition motivational processes related to deliberate practice (Cheng, 2025; Mazlan et al., 2026; Wang et al., 2023).

To address these gaps, the present study proposes a second-stage moderated mediation model linking music learning self-efficacy, achievement goal orientation, AI literacy, and deliberate music practice. Achievement goal orientation is examined as a motivational mechanism in the relationship between music learning self-efficacy and deliberate practice, whereas AI literacy is examined as a boundary condition affecting the strength of the relationship between achievement goal orientation and deliberate practice. This framework allows the study to examine both how competence beliefs are associated with high-quality practice and when this association may be stronger.

The study contributes to the literature by extending research on music learning self-efficacy from performance outcomes to the practice process, integrating achievement goal orientation as a motivational mechanism linking competence beliefs to deliberate practice, and incorporating AI literacy as a technological boundary condition in this motivational pathway. In doing so, the study brings together social-cognitive, achievement-goal, and AI-literacy perspectives within the specific context of deliberate music practice.

## 2. Materials and Methods

### 2.1. Participants and Procedure

Participants were students from the School of Music at a normal university in Liaoning Province, China. The questionnaire was created using Wenjuanxing, an online survey platform, and was distributed in offline classroom settings via a QR code or survey link. Participants were recruited through convenience sampling. Formal data collection was conducted on 4 June 2026. The study was reviewed and approved by the Science and Technology Ethics Committee of Northeast Normal University (approval code: 202602039; approval date: 2 June 2026). Before completing the questionnaire, all participants read an informed consent statement on the first page of the survey. The statement explained the purpose of the study, the voluntary nature of participation, the anonymity of responses, and the confidential use of the data for academic research purposes only. Only participants who provided informed consent were allowed to proceed to the formal questionnaire.

A total of 546 questionnaires were initially collected. To ensure data quality, invalid responses were excluded based on the following criteria: careless or obviously invalid responses, failure to pass the attention check, and a completion time of less than 60 s. After data screening, 458 valid responses were retained for analysis, yielding a valid response rate of 83.88%. Among the participants, 164 were male and 294 were female. Participants ranged in age from 18 to 26 years, with a mean age of 20.54 years (SD = 1.78). The questionnaire included demographic information, music learning background, music learning self-efficacy, deliberate music practice, achievement goal orientation, and AI literacy. Participants also reported their frequency of AI tool use. Only 2.4% reported never using AI tools, whereas 31.0% reported rare use, 30.6% occasional use, 18.1% frequent use, and 17.9% almost daily use. These data provide general contextual information about participants’ exposure to AI, although the specific types of AI tools used and their purposes of use were not assessed in the present study. Detailed sample characteristics are presented in Table 1.

### 2.2. Measures

The questionnaire included measures of music learning self-efficacy, deliberate music practice, achievement goal orientation, and AI literacy. All scales were adapted to the context of music learning where necessary. The number of items, response format, reverse-coded items, and internal consistency reliability of each measure are reported in the following subsections. Before the formal survey, all English-language scales were translated and adapted for the Chinese music learning context using a translation and back-translation procedure. The original English items were first translated into Chinese, after which the Chinese version was independently back-translated into English by a bilingual researcher. The original and back-translated versions were compared, and discrepancies were discussed and revised to improve semantic equivalence and conceptual consistency. The adapted questionnaire was then reviewed by four experts in music education and/or educational psychology for item clarity, relevance, and content appropriateness. Minor wording adjustments were made based on their feedback to improve readability and contextual fit for Chinese music students. A pilot test with 50 music students was subsequently conducted before the formal data collection. Pilot participants provided feedback on item clarity, response format, and completion time. The items were judged to be understandable and suitable for the target sample, and no substantial changes were made after the pilot test.

#### 2.2.1. Music Learning Self-Efficacy

Music learning self-efficacy was measured using the self-efficacy for musical learning subscale developed by Ritchie and Williamon (2011). This scale assesses learners’ confidence in successfully learning and preparing music for a specific performance activity. Participants were asked to recall a recent important musical performance experience and then imagine preparing for a similar performance in the near future. The scale contains 11 items, such as “I am confident that I can successfully learn the music required for this performance.” Responses were rated on a 7-point Likert scale ranging from 1 = not at all sure/strongly disagree to 7 = completely sure/strongly agree. Six items were reverse-coded. Higher scores indicated higher levels of music learning self-efficacy. In the present study, Cronbach’s α for this scale was 0.871.

#### 2.2.2. Deliberate Music Practice

Deliberate music practice was measured using the Deliberate Practice in Music Inventory developed by Passarotto et al. (2022). The inventory was designed to assess the quality of deliberate practice in music learning and performance contexts. It focuses on whether learners engage in goal-directed, reflective, strategic, and problem-solving-oriented practice rather than merely repeating musical materials mechanically. The inventory includes dimensions such as process improvement, practice competences, mindless practice, and task decomposition.

In the present study, the main deliberate practice score was used. The scale contained 23 items. Sample items include “I analyze technical problems,” “I check the effectiveness of the technique I am using,” and “I divide difficult passages into smaller tasks.” Participants responded on a 7-point Likert scale ranging from 1 = never to 7 = always. Six items were reverse-coded. Higher scores indicated a higher level of deliberate music practice. In this study, Cronbach’s α for the scale was 0.927.

#### 2.2.3. Achievement Goal Orientation

Achievement goal orientation was measured using the 12-item Achievement Goal Questionnaire-Revised (AGQ-R) developed by Elliot and Murayama (2008). The original instrument distinguishes four dimensions: mastery-approach, mastery-avoidance, performance-approach, and performance-avoidance goals. In the present study, the items were adapted to the context of music learning. Sample items include “My goal is to learn as much as possible in music learning” and “My goal is to perform better than the other students in music learning.” Participants rated each item on a 5-point Likert scale ranging from 1 = strongly disagree to 5 = strongly agree, and no items were reverse-coded. Because the primary analyses focused on the broader role of achievement-related goal orientation rather than differences among specific goal types, the mean of all 12 items was used as an overall achievement goal orientation score. This aggregation was further evaluated through confirmatory factor analyses comparing one-factor, four-factor correlated, and higher-order factor structures. Higher scores indicated stronger overall achievement goal orientation. Cronbach’s α for the overall score was 0.688, and McDonald’s ω was 0.690.

#### 2.2.4. AI Literacy

AI literacy was measured using the Artificial Intelligence Literacy Scale developed by Wang et al. (2023). This scale assesses individuals’ competence in understanding, using, evaluating, and ethically engaging with AI applications or products. The scale consists of 12 items across four dimensions: awareness, usage, evaluation, and ethics. Sample items include “I can identify the AI technology employed in the applications and products I use” and “I can evaluate the capabilities and limitations of an AI application or product after using it for a while.” Participants responded on a 7-point Likert scale ranging from 1 = strongly disagree to 7 = strongly agree. Three items were reverse-coded. Higher scores indicated a higher level of AI literacy. In the present study, Cronbach’s α for this scale was 0.856.

### 2.3. Data Analysis

Data analyses were conducted using IBM SPSS Statistics 26.0 and IBM SPSS Amos 26.0. Confirmatory factor analysis was performed using Amos 26.0. Mediation and moderated mediation analyses were conducted using the PROCESS macro for SPSS version 4.2 developed by Hayes (2022).

Data analysis was conducted in several steps. First, descriptive statistics were calculated for participants’ demographic information, music learning background, and the main study variables. Second, Cronbach’s α coefficients were calculated to examine the internal consistency of each scale; McDonald’s ω was additionally calculated for the overall achievement goal orientation score. Third, Harman’s single-factor test was conducted to provide a preliminary assessment of potential common method bias (Podsakoff et al., 2003). An item-level confirmatory factor analysis (CFA) including all 58 scale items was then conducted to examine the measurement structure and discriminant validity of music learning self-efficacy (MSE), achievement goal orientation (AGO), AI literacy (AIL), and deliberate music practice (DMP). A four-factor model was compared with a three-factor model in which AI literacy and deliberate music practice were combined and with a one-factor model. Composite reliability (CR), average variance extracted (AVE), and heterotrait–monotrait (HTMT) ratios were also calculated to provide additional evidence regarding reliability, convergent validity, and discriminant validity. Because achievement goal orientation was represented by an overall score in the primary analyses, additional confirmatory factor analyses compared a one-factor model, the original four-factor correlated model, and a higher-order factor model for the 12 AGQ-R items. Model fit was evaluated using the comparative fit index (CFI), Tucker–Lewis index (TLI), root mean square error of approximation (RMSEA), and standardized root mean square residual (SRMR) (Hu & Bentler, 1999; Kline, 2016).

Pearson correlation analysis was then used to examine the associations among the main variables. Finally, the mediation and moderated mediation effects were tested using the PROCESS macro developed by Hayes (2022). No custom computer code was developed; all analyses were conducted using the software and macro described above. Specifically, PROCESS Model 4 was used to test whether achievement goal orientation mediated the relationship between music learning self-efficacy and deliberate music practice. PROCESS Model 14 was then used to test the second-stage moderated mediation model, in which music learning self-efficacy was entered as the independent variable, achievement goal orientation as the mediator, deliberate music practice as the dependent variable, and AI literacy as the moderator of the path from achievement goal orientation to deliberate music practice.

The significance of indirect effects was tested using the bootstrap method with 5000 resamples (Preacher & Hayes, 2008). An indirect effect was considered significant when the 95% bootstrap confidence interval did not include zero. For the moderation effect, the interaction between achievement goal orientation and AI literacy was examined, and the incremental change in explained variance (ΔR^2^) attributable to the interaction was reported. Simple slope and Johnson–Neyman analyses were used to interpret the conditional association between achievement goal orientation and deliberate music practice across levels of AI literacy. Multicollinearity was evaluated using tolerance and variance inflation factor (VIF) statistics. Finally, sensitivity analyses repeated the proposed model while controlling for grade, major, years of systematic music study, practice frequency, and AI-use frequency. Grade and major were dummy-coded, while years of music study, practice frequency, and AI-use frequency were entered as ordered covariates.

## 3. Results

### 3.1. Common Method Bias and Confirmatory Factor Analysis

Because all variables were collected using self-report questionnaires, Harman’s single-factor test was conducted to examine potential common method bias (Podsakoff et al., 2003). The first unrotated factor explained 29.69% of the total variance, below the commonly used threshold of 40%. This result provided preliminary evidence regarding common method bias; however, Harman’s single-factor test alone cannot rule out potential common method effects.

An item-level confirmatory factor analysis was conducted using all 58 items. As shown in Table 2, the four-factor model showed good fit, χ^2^ = 1789.203, df = 1589, χ^2^/df = 1.126, CFI = 0.976, TLI = 0.975, RMSEA = 0.017, SRMR = 0.035. This model fitted significantly better than the three-factor model in which AI literacy and deliberate music practice were combined, Δχ^2^ = 36.419, Δdf = 3, *p* < 0.001, and also fitted better than the one-factor model. These results supported the four-factor measurement structure. However, the latent correlation between AI literacy and deliberate music practice was high (r = 0.943), indicating that the discriminant validity of these two constructs should be interpreted cautiously (Table 3).

Composite reliability values ranged from 0.688 to 0.927. AVE values ranged from 0.160 to 0.385 and were below the conventional 0.50 criterion, suggesting that convergent validity should be interpreted cautiously, particularly for achievement goal orientation. HTMT ratios ranged from 0.730 to 0.942; the AI literacy–deliberate music practice ratio was 0.942, exceeding 0.90 and indicating substantial overlap between these constructs. Multicollinearity diagnostics for the moderated mediation model did not indicate severe multicollinearity: tolerance/VIF values were 0.497/2.012 for music learning self-efficacy, 0.559/1.789 for achievement goal orientation, 0.426/2.348 for AI literacy, and 0.963/1.038 for the AGO × AIL interaction term.

### 3.2. Additional Structure Check for Achievement Goal Orientation

Because the present study used an overall achievement goal orientation score, additional confirmatory factor analyses were conducted to examine whether the 12 AGQ-R items could reasonably support an overall representation. The one-factor model showed good fit, χ^2^ = 68.441, df = 54, χ^2^/df = 1.267, CFI = 0.966, TLI = 0.959, RMSEA = 0.024, SRMR = 0.041. The four-factor correlated model also showed good fit, χ^2^ = 63.439, df = 48, χ^2^/df = 1.322, CFI = 0.964, TLI = 0.950, RMSEA = 0.027, SRMR = 0.039, and the higher-order factor model similarly fitted the data well, χ^2^ = 65.229, df = 50, χ^2^/df = 1.305, CFI = 0.964, TLI = 0.953, RMSEA = 0.026, SRMR = 0.040.

Correlations among the four first-order dimensions ranged from 0.727 to 0.970. The overall achievement goal orientation score showed modest but comparable internal consistency across Cronbach’s α (0.688) and McDonald’s ω (0.690). Taken together, these results provided empirical support for using the overall achievement goal orientation score as a parsimonious summary measure in the present analyses, while retaining the conceptual distinction among the original goal dimensions.

### 3.3. Descriptive Statistics and Correlations

Descriptive statistics, reliability coefficients, and correlations among the main variables are presented in Table 4. Cronbach’s α coefficients ranged from 0.688 to 0.927, with the overall achievement goal orientation score showing comparatively modest internal consistency (α = 0.688; ω = 0.690). Music learning self-efficacy was positively correlated with achievement goal orientation, AI literacy, and deliberate music practice. Achievement goal orientation was also positively correlated with AI literacy and deliberate music practice. AI literacy showed a strong positive correlation with deliberate music practice (r = 0.842), consistent with the measurement-model results indicating substantial overlap between these two constructs. These findings should therefore be interpreted with appropriate caution.

### 3.4. Testing the Mediation and Moderated Mediation Model

The results of the mediation and moderated mediation analyses are presented in Table 5. In Model 1, music learning self-efficacy was significantly and positively associated with achievement goal orientation, β = 0.565, t = 14.634, *p* < 0.001. This result indicated that learners with higher music learning self-efficacy tended to report stronger achievement goal orientation.

In Model 2, music learning self-efficacy was significantly and positively associated with deliberate music practice, β = 0.500, t = 15.000, *p* < 0.001. This finding indicated that learners with higher music learning self-efficacy tended to report higher levels of deliberate music practice. Therefore, H1 was supported.

In the same model, achievement goal orientation was also significantly and positively associated with deliberate music practice, β = 0.415, t = 12.444, *p* < 0.001. Together with the significant association between music learning self-efficacy and achievement goal orientation in Model 1, these results provided preliminary evidence for the mediating role of achievement goal orientation. The bootstrap analysis further showed that the indirect association between music learning self-efficacy and deliberate music practice through achievement goal orientation was significant, indirect effect = 0.218, 95% CI [0.174, 0.261]. Therefore, H2 was supported.

In Model 3, AI literacy was entered as the moderator of the path from achievement goal orientation to deliberate music practice. Music learning self-efficacy remained significantly and positively associated with deliberate music practice, β = 0.259, t = 8.401, *p* < 0.001. Achievement goal orientation also remained positively associated with deliberate music practice, β = 0.217, t = 7.438, *p* < 0.001. AI literacy showed a significant positive association with deliberate music practice, β = 0.520, t = 15.585, *p* < 0.001.

More importantly, the interaction between achievement goal orientation and AI literacy was significant, β = 0.068, t = 2.531, *p* = 0.012. The interaction accounted for a small but statistically significant increment in explained variance, ΔR^2^ = 0.0030, Fchange = 6.407, *p* = 0.012. Thus, AI literacy significantly moderated the relationship between achievement goal orientation and deliberate music practice, although the incremental effect size was small. Therefore, H3 was supported.

**Figure 1:**
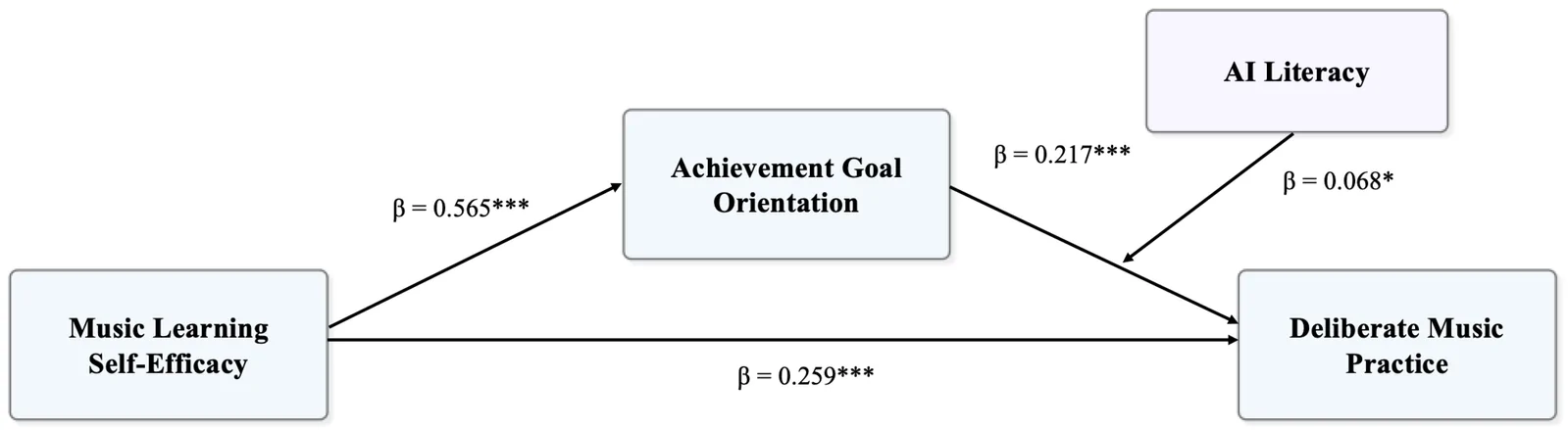
Moderated mediation model of music learning self-efficacy, achievement goal orientation, AI literacy, and deliberate music practice. * *p* < 0.05; *** *p* < 0.001.

The overall moderated mediation model is shown in Figure 1.

### 3.5. Conditional Indirect Effects

To further examine the moderated mediation effect, conditional indirect effects were tested at low, mean, and high levels of AI literacy. As shown in Table 6, the indirect effect of music learning self-efficacy on deliberate music practice through achievement goal orientation was significant at all three levels of AI literacy.

Specifically, when AI literacy was low, the indirect effect was significant, effect = 0.083, Boot SE = 0.027, 95% CI [0.031, 0.136]. At the mean level of AI literacy, the indirect effect was also significant, effect = 0.122, Boot SE = 0.020, 95% CI [0.083, 0.163]. When AI literacy was high, the indirect effect became stronger, effect = 0.161, Boot SE = 0.027, 95% CI [0.110, 0.217].

The index of moderated mediation was 0.039, with a 95% bootstrap confidence interval of [0.004, 0.075]. Because the confidence interval did not include zero, the moderated mediation effect was significant. These results indicated that the indirect relationship between music learning self-efficacy and deliberate music practice through achievement goal orientation became stronger as AI literacy increased. Therefore, H4 was supported.

### 3.6. Johnson–Neyman Analysis

**Figure 2:**
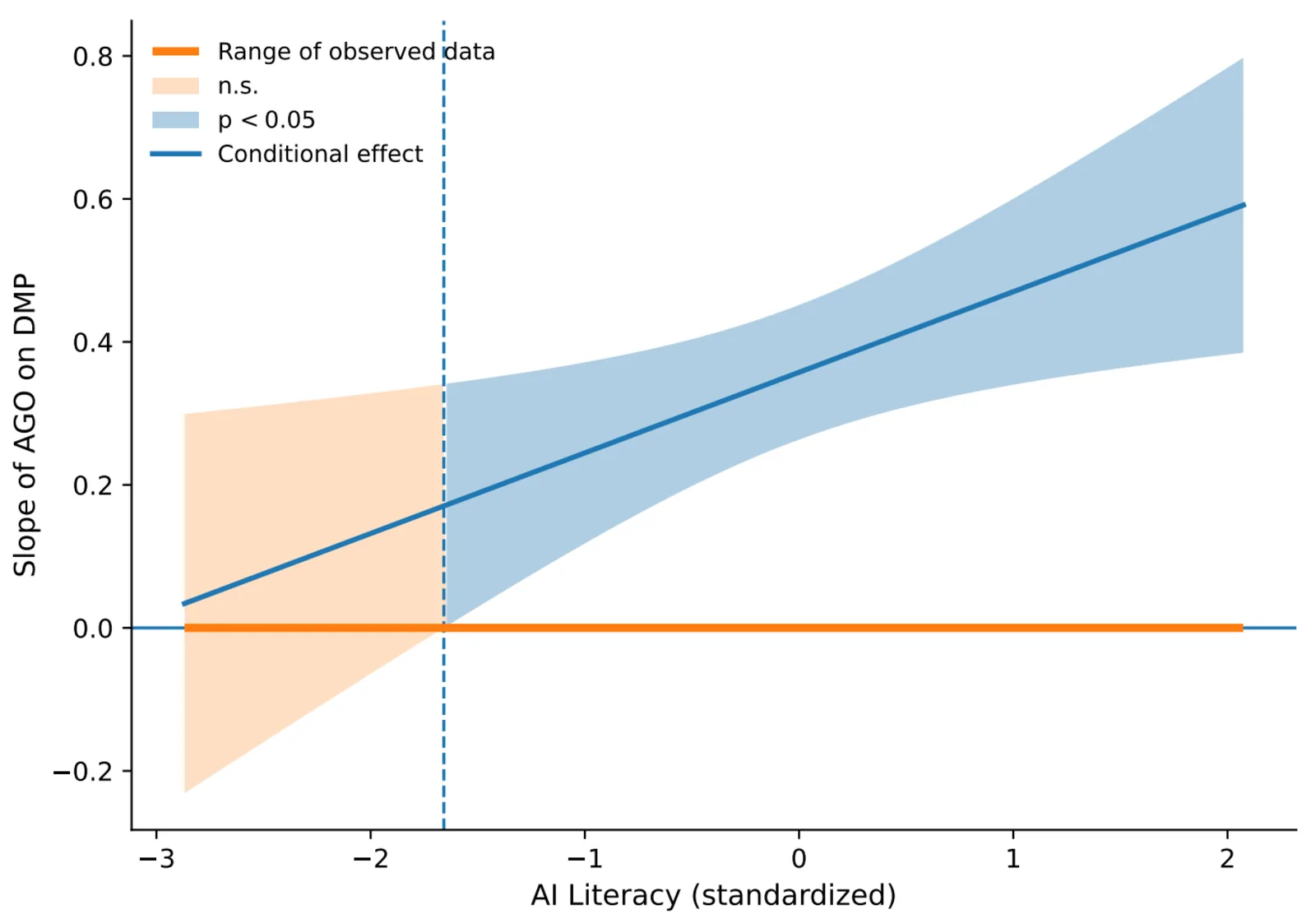
Johnson–Neyman plot of the moderating effect of AI literacy on the relationship between achievement goal orientation and deliberate music practice. The blue dashed vertical line indicates the Johnson–Neyman significance threshold at AI literacy = −1.66 SD.

To further examine the moderating effect of AI literacy, a Johnson–Neyman analysis was conducted. As shown in Figure 2, the conditional association between achievement goal orientation and deliberate music practice increased as AI literacy increased. The association became statistically significant when standardized AI literacy exceeded −1.66 SD, corresponding to an original-scale AI literacy score of approximately 3.52. This result further supports the moderating role of AI literacy while providing the numerical region of significance for the interaction.

### 3.7. Sensitivity Analyses

Sensitivity analyses repeated the moderated mediation model while controlling for grade, major, years of systematic music study, practice frequency, and AI-use frequency. The substantive results remained unchanged. Music learning self-efficacy remained positively associated with achievement goal orientation (β = 0.250, t = 4.748, *p* < 0.001) and deliberate music practice (β = 0.165, t = 5.141, *p* < 0.001). Achievement goal orientation (β = 0.176, t = 6.179, *p* < 0.001) and AI literacy (β = 0.416, t = 11.774, *p* < 0.001) remained positively associated with deliberate music practice, and the AGO × AIL interaction remained significant (β = 0.072, t = 2.795, *p* = 0.005; ΔR^2^ = 0.0033, Fchange = 7.814, *p* = 0.005).

The conditional indirect association also remained significant at low AI literacy (effect = 0.026, Boot SE = 0.011, 95% CI [0.005, 0.051]), mean AI literacy (effect = 0.044, Boot SE = 0.013, 95% CI [0.022, 0.071]), and high AI literacy (effect = 0.062, Boot SE = 0.019, 95% CI [0.029, 0.104]). The index of moderated mediation remained significant, index = 0.018, Boot SE = 0.010, 95% CI [0.002, 0.040]. In the covariate-adjusted model, the Johnson–Neyman threshold was −1.28 SD, corresponding to an original-scale AI literacy score of approximately 3.84. These sensitivity analyses indicate that the main conclusions were robust to adjustment for the selected background variables.

## 4. Discussion

The present study examined the relationships underlying deliberate music practice among music learners, focusing on music learning self-efficacy, achievement goal orientation, and AI literacy. Music learning self-efficacy was positively associated with deliberate music practice, with a significant indirect association through achievement goal orientation. AI literacy further moderated the association between achievement goal orientation and deliberate music practice, and the conditional indirect effect was stronger among learners with higher levels of AI literacy. Overall, these findings suggest that high-quality music practice is related not only to practice conditions but also to learners’ competence beliefs, achievement-related goals, and AI-related competence.

### 4.1. Music Learning Self-Efficacy and Deliberate Music Practice

The first finding of this study was that music learning self-efficacy was positively associated with deliberate music practice. This result suggests that whether music learners engage in high-quality practice is related not only to the practice task itself, but also to how they perceive their own capabilities. For music learners, difficulties encountered during practice are not merely abstract learning pressures; they often take the form of concrete problems involving difficult passages, technical movements, rhythmic control, intonation, musical understanding, and performance preparation. Learners with higher music learning self-efficacy may be more likely to interpret these difficulties as problems that can be gradually improved through sustained practice and strategic adjustment, rather than as evidence of insufficient ability.

This finding deepens the understanding of deliberate music practice. Deliberate music practice emphasizes clear goals, problem diagnosis, feedback use, and strategic adjustment, requiring learners to engage in a continuous cycle of identifying problems, making revisions, and re-evaluating progress (Ericsson et al., 1993; Passarotto et al., 2022). This process can be demanding because it requires learners to repeatedly confront errors, recognize that their current practice methods may be ineffective, and invest additional effort in making adjustments. The role of music learning self-efficacy may lie in providing psychological support for learners to engage in this demanding practice process. In other words, learners with higher self-efficacy are not simply “more confident”; they may also be more likely to believe that problems encountered during practice can be improved and, therefore, more willing to engage in conscious and strategic practice.

This finding also extends research on musical self-efficacy from the outcome level to the process level. Previous studies of musical self-efficacy have mainly focused on its relationship with music performance, performance examinations, or learning outcomes (McCormick & McPherson, 2003; McPherson & McCormick, 2006). The present study further suggests that music learning self-efficacy is associated with the quality of practice preceding performance. This shift is important because musical performance develops through sustained practice processes rather than through self-efficacy alone. If research focuses only on final performance, it may overlook how learners engage, revise, and reflect during practice. By treating deliberate music practice as the outcome variable, the present study provides a more process-oriented understanding of the role of self-efficacy in music learning.

From a practical perspective, this finding also suggests that improving practice quality should not rely solely on increasing practice time or emphasizing practice discipline. If learners lack a basic belief in their own music learning capabilities, they may complete practice tasks while remaining at the level of mechanical repetition or passive task completion. In contrast, when learners believe that they can improve through practice, they may be more likely to analyze problems, try different strategies, and evaluate the effectiveness of their practice. Therefore, when guiding music practice, teachers need not only to tell students what and how to practice, but also to help them develop the belief that they can improve through effective practice by providing specific feedback, staged goals, and visible experiences of progress.

Taken together, the support for H1 indicates that music learning self-efficacy is an important psychological variable associated with deliberate music practice. It is related not only to learners’ willingness to engage in music learning, but also to whether they report organizing their practice in a goal-directed, problem-focused, and strategically regulated manner. This finding provides a foundation for considering achievement goal orientation as a possible motivational link between competence beliefs and clearer practice directions.

### 4.2. The Mediating Role of Achievement Goal Orientation

The present study found a significant indirect association between music learning self-efficacy and deliberate music practice through achievement goal orientation. Higher music learning self-efficacy was associated with stronger achievement goal orientation, which in turn was related to more directed and strategic practice behaviors. In this sense, achievement goal orientation may help explain how competence beliefs are linked to clearer practice purposes and learning directions, without implying a causal sequence based on the present cross-sectional data.

This result is consistent with the connection between social cognitive theory and achievement goal theory. According to social cognitive theory, self-efficacy is associated with how learners approach tasks, sustain effort, and regulate their behavior (Bandura, 1997), while achievement goals are related to how learners understand success, effort, and strategy use in learning (Ames, 1992; Pintrich, 2000). In music learning, this connection is particularly relevant because deliberate practice requires goal setting, problem analysis, progress monitoring, and strategic adjustment rather than simple repetition (Passarotto et al., 2022). Achievement goal orientation may therefore help explain the association between music learning self-efficacy and concrete practice behaviors.

This finding also extends previous research. Earlier studies have linked self-efficacy to music performance and learning outcomes (McCormick & McPherson, 2003; McPherson & McCormick, 2006), while research on music practice has emphasized planning, monitoring, and strategic regulation as features of effective practice (Hallam, 2001; Miksza, 2011a). The present study connects these two lines of research by identifying achievement goal orientation as a plausible motivational pathway associated with the relationship between music learning self-efficacy and deliberate music practice. Learners who report stronger competence beliefs may also report clearer achievement-related goals, which in turn are associated with more purposeful practice.

From a practical perspective, this finding suggests that music teachers can help students connect confidence with specific practice goals. For example, a broad intention such as “I want to play this piece well” can be refined into more concrete goals, such as improving rhythmic accuracy, addressing a technical difficulty, or refining the expression of a specific phrase. Such goal clarification may support more purposeful and strategy-oriented practice. Overall, the support for H2 suggests that achievement goal orientation may represent an important motivational link between music learning self-efficacy and deliberate music practice.

### 4.3. AI Literacy as a Boundary Condition

The present study found that AI literacy moderated the relationship between achievement goal orientation and deliberate music practice. Specifically, the positive association between achievement goal orientation and deliberate music practice was stronger when AI literacy was higher. This pattern suggests that AI literacy may be associated with how effectively learners connect achievement-related goals with concrete, adjustable practice strategies.

This result is meaningful in the context of AI-supported music learning. Deliberate music practice requires learners to identify problems, obtain feedback, compare strategies, and continuously revise the practice process (Passarotto et al., 2022). AI tools may support these processes by helping learners retrieve music knowledge, analyze musical structure, generate practice suggestions, or receive feedback (Mazlan et al., 2026; Merchán Sánchez-Jara et al., 2024). However, AI tools themselves do not automatically improve practice quality. Their usefulness depends on whether learners can understand what AI can and cannot do, evaluate the reliability of AI-generated outputs, and adapt AI suggestions to their own practice contexts (Long & Magerko, 2020; Wang et al., 2023).

In this sense, AI literacy can be understood as a form of resource-use competence. Learners with stronger achievement goal orientation may already report clearer motivation to improve, but the extent to which this motivation is associated with deliberate practice may differ according to their ability to use AI resources. Learners with higher AI literacy may be better positioned to use AI tools to clarify practice goals, break down difficult tasks, compare possible strategies, and evaluate feedback. By contrast, learners with lower AI literacy may be less able to use such resources effectively or may be more vulnerable to misusing AI-generated feedback. This interpretation is consistent with the finding that the association between achievement goal orientation and deliberate practice was stronger at higher levels of AI literacy.

The moderated mediation results further indicated that the indirect association between music learning self-efficacy and deliberate music practice through achievement goal orientation varied across levels of AI literacy. Higher music learning self-efficacy was associated with stronger achievement goal orientation, which was in turn associated with more deliberate practice, and this latter association was stronger among learners reporting higher AI literacy. These findings therefore extend a purely motivational account of deliberate music practice by suggesting that the association between motivational factors and practice behavior may depend partly on learners’ AI-related competence.

Overall, the support for H3 and H4 indicates that AI literacy is a relevant boundary condition in the proposed model. However, the interaction accounted for only a small increment in explained variance (ΔR^2^ = 0.0030). AI literacy should therefore be interpreted as a modest rather than strong boundary condition: the association between achievement goal orientation and deliberate practice was statistically stronger at higher levels of AI literacy, but the practical magnitude of this moderation was limited.

### 4.4. Theoretical Contributions and Practical Implications

The present study makes three theoretical contributions. First, it extends research on deliberate music practice from describing the characteristics of effective practice to examining learner-level motivational correlates of such practice. Previous research has shown that deliberate music practice involves goal setting, problem diagnosis, feedback use, and strategic adjustment (Ericsson et al., 1993; Passarotto et al., 2022). By treating deliberate music practice itself, rather than performance outcomes, as the focal outcome, the present study shifts attention toward the practice process and shows that learners’ competence beliefs and achievement-related motivation are closely associated with whether they report engaging in high-quality practice.

Second, the study integrates social cognitive theory and achievement goal theory within the specific context of music learning. Rather than examining self-efficacy only as a correlate of performance outcomes, the present model positions achievement goal orientation as a motivational link between music learning self-efficacy and deliberate practice. This helps connect research on competence beliefs with research on planning, monitoring, and strategic regulation in music practice, thereby providing a more explicit account of how these two motivational traditions can be brought together (Bandura, 1997; Pintrich, 2000).

Third, the study extends this motivational framework by incorporating AI literacy as a technological boundary condition. Existing research on AI in education has often emphasized the functions, opportunities, and risks of AI tools (Chiu et al., 2023; Zawacki-Richter et al., 2019). The present findings add that the association between achievement goal orientation and deliberate practice was stronger among learners with higher AI literacy. This positions AI literacy as a learning-related competence that may condition how strongly motivational resources are associated with goal-directed practice in AI-supported learning environments.

The findings also have implications for music education. Music teachers can support deliberate practice by helping students develop confidence in their ability to improve through effective practice and by guiding them to translate broad learning intentions into concrete, manageable goals. Specific feedback, staged goals, and visible progress may strengthen music learning self-efficacy, while goal clarification can help students focus on particular technical, rhythmic, or expressive problems and organize practice more purposefully.

In AI-supported music learning environments, educators may also consider developing students’ critical and strategic AI literacy rather than focusing only on technical access or tool operation. Potential instructional applications include helping students recognize the capabilities and limitations of AI systems, evaluate the accuracy and relevance of AI-generated suggestions, compare AI feedback with teacher feedback and musical evidence, select AI use that is appropriate to the practice task, and adapt rather than passively accept AI-generated recommendations. These suggestions should be understood as potential educational applications rather than practices directly tested in the present study, because the study measured general AI literacy rather than students’ actual use of AI during music practice.

### 4.5. Limitations and Future Directions

Several limitations should be noted. First, the present study used a cross-sectional questionnaire design. Although the results supported the proposed mediation and moderated mediation model, causal relationships among music learning self-efficacy, achievement goal orientation, AI literacy, and deliberate music practice cannot be inferred. Future research could use longitudinal designs to examine whether changes in music learning self-efficacy predict later changes in achievement goal orientation and deliberate music practice. Experimental or intervention studies could also be conducted to test whether improving learners’ self-efficacy or AI literacy can lead to higher-quality practice behaviors.

Second, all variables were measured using self-report questionnaires. Although Harman’s single-factor test provided preliminary evidence regarding common method bias, this procedure alone cannot rule out potential common method effects. Self-report data may also be influenced by social desirability, memory bias, or learners’ subjective understanding of their own practice behaviors. Future studies could combine self-report measures with teacher ratings, practice logs, behavioral observations, or digital records of practice activities. Such multi-source data would provide a more objective understanding of deliberate music practice.

An additional measurement limitation concerns the strong association between AI literacy and deliberate music practice. Although the item-level four-factor CFA fitted significantly better than a model combining these constructs, their latent correlation was 0.943 and the AIL–DMP HTMT ratio was 0.942. In addition, AVE values for the study constructs were below 0.50. These results suggest that the discriminant and convergent validity of the measures should be interpreted cautiously. Future research should use more differentiated, music-specific, and behavioral measures to clarify the conceptual boundaries between AI-related competence and deliberate practice behavior.

Third, the sample was drawn from students in the School of Music at one normal university in Liaoning Province, China. This sample was appropriate for examining the proposed model among music learners, but the generalizability of the findings may be limited. Future research could include students from conservatories, comprehensive universities, and different regions, as well as learners with different levels of musical expertise. Cross-cultural studies may also help examine whether the role of AI literacy in deliberate music practice varies across educational and technological contexts.

Fourth, the primary analyses used an overall achievement goal orientation score. Additional confirmatory factor analyses showed that the one-factor, four-factor correlated, and higher-order models all demonstrated acceptable fit, while the high correlations among the four first-order dimensions provided empirical support for using an overall score in the present analyses. Nevertheless, the original AGQ-R distinguishes mastery-approach, mastery-avoidance, performance-approach, and performance-avoidance goals as conceptually distinct dimensions, and the reliability of the overall score was comparatively modest (Cronbach’s α = 0.688; McDonald’s ω = 0.690). Future research could therefore examine whether these specific goal types show distinct relationships with music learning self-efficacy and deliberate music practice.

Finally, AI literacy was measured as a general competence in understanding, using, evaluating, and ethically engaging with AI. Although participants reported their overall frequency of AI tool use, the study did not collect detailed information about the specific AI tools they used or the purposes for which they used them. This limits the contextual interpretation of how AI literacy operates in actual music learning activities. Future research could record the types of AI tools used, their purposes and task contexts, and learners’ patterns of use, while also developing music-specific AI literacy measures that capture abilities such as evaluating AI-generated performance feedback, using AI for music analysis, and integrating AI suggestions into practice planning.

## 5. Conclusions

The present study examined how music learning self-efficacy, achievement goal orientation, and AI literacy are associated with deliberate music practice. Music learning self-efficacy was positively associated with deliberate music practice, and a significant indirect association through achievement goal orientation was observed. In addition, the positive association between achievement goal orientation and deliberate music practice was stronger among learners with higher levels of AI literacy, and the corresponding indirect association between music learning self-efficacy and deliberate music practice also varied across levels of AI literacy. These findings reflect statistical associations based on cross-sectional self-report data and should not be interpreted as evidence of causal relationships.

The data therefore support associations among music learning self-efficacy, the overall achievement goal orientation score, AI literacy, and self-reported deliberate music practice. The educational implications proposed here should be viewed as potential applications of these associations rather than as evidence that AI literacy training or specific AI-supported practice strategies improve practice quality. Future research using longitudinal, experimental, behavioral, and music-specific AI literacy designs is needed to examine the temporal and causal processes suggested by the present model.

## Figures and Tables

**Table 1:** Sample characteristics (N = 458).

Variable	Category	*n*	%
Gender	Male	164	35.8
	Female	294	64.2
Grade	Freshman	137	29.9
	Sophomore	86	18.8
	Junior	94	20.5
	Senior	103	22.5
	Master’s student	38	8.3
Major	Music performance	146	31.9
	Music education	119	26.0
	Composition and compositional theory	68	14.8
	Musicology	44	9.6
	Music technology/production	51	11.1
	Dance/performing arts-related major	30	6.6
Years of systematic music learning	1–3 years	217	47.4
	4–6 years	223	48.7
	7–10 years	18	3.9
Weekly music practice frequency	Less than once per week	25	5.5
	1–2 times per week	98	21.4
	3–4 times per week	128	27.9
	5–6 times per week	102	22.3
	Every day or almost every day	105	22.9
AI tool use frequency	Never used	11	2.4
	Used, but rarely	142	31.0
	Occasionally used	140	30.6
	Frequently used	83	18.1
	Almost daily	82	17.9

Note. AI = artificial intelligence.

**Table 2:** Confirmatory factor analysis model fit indices.

Model	χ^2^	df	χ^2^/df	CFI	TLI	RMSEA	SRMR
Four-factor item-level model	1789.203	1589	1.126	0.976	0.975	0.017	0.035
Three-factor model (AIL + DMP combined)	1825.622	1592	1.147	0.971	0.970	0.018	0.035
One-factor item-level model	2175.413	1595	1.364	0.929	0.926	0.028	0.040

Note. Confirmatory factor analysis was conducted at the item level using all 58 items. CFI = comparative fit index; TLI = Tucker–Lewis index; RMSEA = root mean square error of approximation; SRMR = standardized root mean square residual; AIL = AI Literacy; DMP = Deliberate Music Practice.

**Table 3:** Composite reliability, average variance extracted, and HTMT ratios.

Construct	CR	AVE	1	2	3	4
1. MSE	0.873	0.385	–			
2. AGO	0.688	0.160	0.730	–		
3. AIL	0.857	0.336	0.790	0.837	–	
4. DMP	0.927	0.357	0.817	0.875	0.942	–

Note. Values below the diagonal are HTMT ratios. HTMT = heterotrait–monotrait ratio; CR = composite reliability; AVE = average variance extracted; MSE = Music Learning Self-Efficacy; AGO = Achievement Goal Orientation; AIL = AI Literacy; DMP = Deliberate Music Practice.

**Table 4:** Descriptive statistics, reliability, and correlations (N = 458).

Variables	M	SD	α	1	2	3	4
1. MSE	4.88	0.88	0.871	–			
2. AGO	3.65	0.50	0.688	0.565 ***	–		
3. AIL	4.92	0.84	0.856	0.682 ***	0.640 ***	–	
4. DMP	4.96	0.82	0.927	0.734 ***	0.697 ***	0.842 ***	–

Note. MSE = Music Learning Self-Efficacy; AGO = Achievement Goal Orientation; AIL = AI Literacy; DMP = Deliberate Music Practice. *** *p* < 0.001.

**Table 5:** Results of the moderated mediation model.

Predictor	Outcome: AGO (Model 1)			Outcome: DMP (Model 2)			Outcome: DMP (Model 3)		
	β	t	*p*	β	t	*p*	β	t	*p*
MSE	0.565	14.634	<0.001	0.500	15.000	<0.001	0.259	8.401	<0.001
AGO				0.415	12.444	<0.001	0.217	7.438	<0.001
AIL							0.520	15.585	<0.001
AGO × AIL							0.068	2.531	0.012
R^2^	0.320			0.656			0.785		
F	214.168			434.233			414.349		

Note. Bootstrap sample = 5000. All variables were standardized before analysis. MSE = Music Learning Self-Efficacy; AGO = Achievement Goal Orientation; AIL = AI Literacy; DMP = Deliberate Music Practice. The interaction term was calculated using standardized AGO and AIL.

**Table 6:** Conditional indirect effect of music learning self-efficacy on deliberate music practice via achievement goal orientation.

Moderator: AI Literacy	AIL Level	Effect	Boot SE	LLCI	ULCI
AI literacy	Low (M − 1 SD)	0.083	0.027	0.031	0.136
	Mean	0.122	0.020	0.083	0.163
	High (M + 1 SD)	0.161	0.027	0.110	0.217
Index of moderated mediation		0.039	0.018	0.004	0.075

Note. Bootstrap sample = 5000. All variables were standardized before analysis. Boot SE = bootstrap standard error; LLCI = lower limit confidence interval; ULCI = upper limit confidence interval.

## Data Availability

The data presented in this study are available from the corresponding author upon reasonable request due to privacy and ethical restrictions.
